# Varicella-Zoster Virus: A Case of Encephalitis

**DOI:** 10.7759/cureus.45378

**Published:** 2023-09-16

**Authors:** Filipa Rodrigues, Mariana Santos, Eduardo Macedo, José Miguel Rocha, Luís Silva, Ana S Oliveira, Joana Alves, Isabel Apolinário

**Affiliations:** 1 Internal Medicine, Hospital de Braga, Braga, PRT; 2 Neuroradiology, Hospital de Braga, Braga, PRT; 3 Oncology, Hospital de Braga, Braga, PRT; 4 Infectious Disease, Hospital de Braga, Braga, PRT

**Keywords:** varicella-zoster virus, diplopia, human immunodeficiency virus, central nervous system infection, case report, encephalitis

## Abstract

Infection with the varicella-zoster virus (VZV) is very common worldwide and is one of the main causes of infectious encephalitis. Immunosuppressed patients are at increased risk of severe disease and central nervous system (CNS) involvement. We report the case of a 43-year-old man with HIV infection and poor adherence to antiretroviral therapy who presented to the emergency department (ER) with complaints of diplopia and a frontal headache, referring to having a child with chickenpox. Brain magnetic resonance imaging revealed three hyperintense T2-weighted lesions surrounded by edema in the right sublenticular, left occipital and left parietal regions, and VZV DNA was detected in the cerebrospinal fluid (CSF). After admitting the diagnosis of VZV encephalitis, the patient was treated with intravenous acyclovir, with clinical improvement and a favorable outcome.

## Introduction

The varicella-zoster virus (VZV) is a virus of the alpha herpesvirus family that is neurotropic and has double-stranded DNA. The first contact with this virus usually occurs in childhood, and the primary infection causes chickenpox, during which the VZV becomes latent in the cranial nerve, dorsal root, and autonomic ganglia along the entire neuraxis [1,2].

Since the immune control of the virus is mainly mediated by T cells, reactivation usually occurs with aging or as a consequence of immunosuppression and manifests as zoster, which is typically characterized by a vesicular rash in the dermatomal distribution of the sensory ganglion and acute neuritis [3].

VZV is one of the main causes of infectious neurological diseases, being the second most common cause of encephalitis and infectious meningitis, with a mortality rate of 12% to 15%, which may be higher in immunosuppressed patients [4]. The diagnosis is based on clinical manifestations, neurological symptoms, an analysis of cerebrospinal fluid (CSF), and imaging abnormalities. The treatment involves the administration of antivirals, which can lead to a complete recovery [2].

## Case presentation

A 43-year-old male construction worker with a history of hepatitis C treated 10 years ago and infection with the human immunodeficiency virus (HIV) for 20 years, under antiretroviral therapy (ART), with poor adherence to therapy and a history of opportunistic infections, namely Pneumocystis jiroveci pneumonia and esophageal candidiasis for about five months, was presented to the emergency department.

He was presented to the emergency department due to diplopia with three weeks of evolution, having been observed by neurology, which showed paresis of the III left cranial nerve without other neurological alterations, and asked for cranial computed tomography (CT), which did not reveal alterations, so, despite doubts about the etiology of this clinical alteration, he medicated with platelet antiaggregant and was referred for external consultation. As he continued to complain, three days later he went back to the ED, associating a frontal headache with the same evolution time. He denied skin changes, fever, eye pain, eye injury, hypovision, or traumatic brain injury. He mentioned that his son had been infected with the chickenpox virus for at least four days, and the patient did not know if he had had this disease in childhood. On physical examination, he was apyretic, hemodynamically stable, maintaining diplopia and paresis of the III cranial nerve, without motor or gait alterations, and without skin alterations. Kernig and Brudzinski's signs were negative. He was observed by ophthalmology, which ruled out lesions suggestive of retinitis. His cranial and orbital CTs did not show changes. He also performed a lumbar puncture with clear liquid output, lymphocytic pleocytosis (37 cells, 88% lymphocytes), proteinorrachia (0.49 g/L), normal glucose, a negative venereal disease research laboratory (VDRL) test, treponema pallidum haemagglutination (TPHA), antimicrobial microbiological and positive VZV DNA research, and negative herpes simplex virus DNA research.

**Figure 1:**
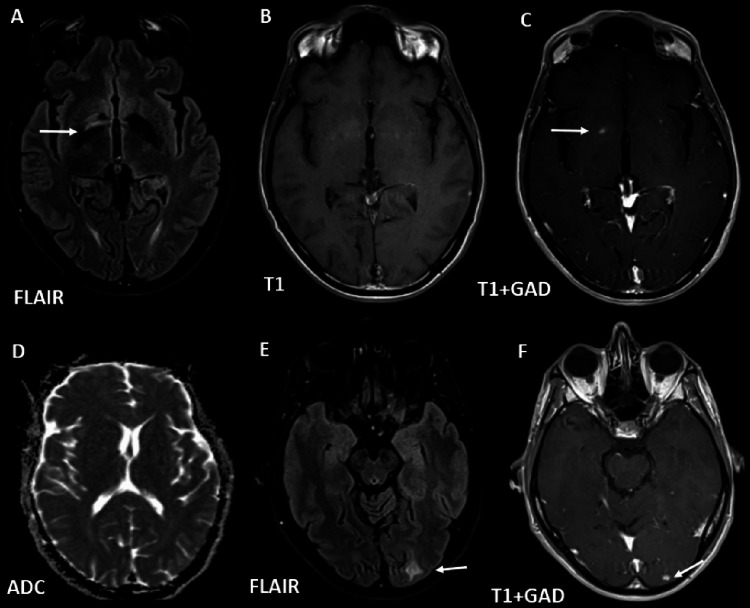
Brain magnetic resonance imaging Axial FLAIR image showing lesion with hypersignal in the right sublenticular region (A, arrow), not perceptible on T1-weighted images (B), demonstrating nodular enhancement after contrast (C, arrow), without diffusion restriction (D), FLAIR axial images (E, arrow) revealing a left occipital subcortical white matter lesion with post-contrast nodular enhancement (F, arrow). FLAIR: fluid-attenuated inversion recovery

The patient was hospitalized to complete the study and start treatment. Analytically, without alterations in blood count or renal function, without cytocholestasis, without alterations in coagulation, with a negative C-reactive protein, highlighting a CD4 cell count of 64/uL. A brain magnetic resonance imaging (MRI) was performed, demonstrating a small nodular lesion in the right sublenticular region with hyperintense signal on the axial T2-weighted image and homogeneous enhancement on the axial T1-weighted image after gadolinium, surrounded by edema. In the left occipital and parietal regions, involving the juxtacortical region, two other similar lesions can be observed (Figure 1). A diagnosis of encephalitis due to the varicella-zoster virus was admitted, and he started aciclovir 800 mg every eight hours, which he fulfilled for 14 days. The patient remained hemodynamically stable during hospitalization and with improvement of the deficits described, maintaining only diplopia in the extreme look to the left.

**Figure 2:**
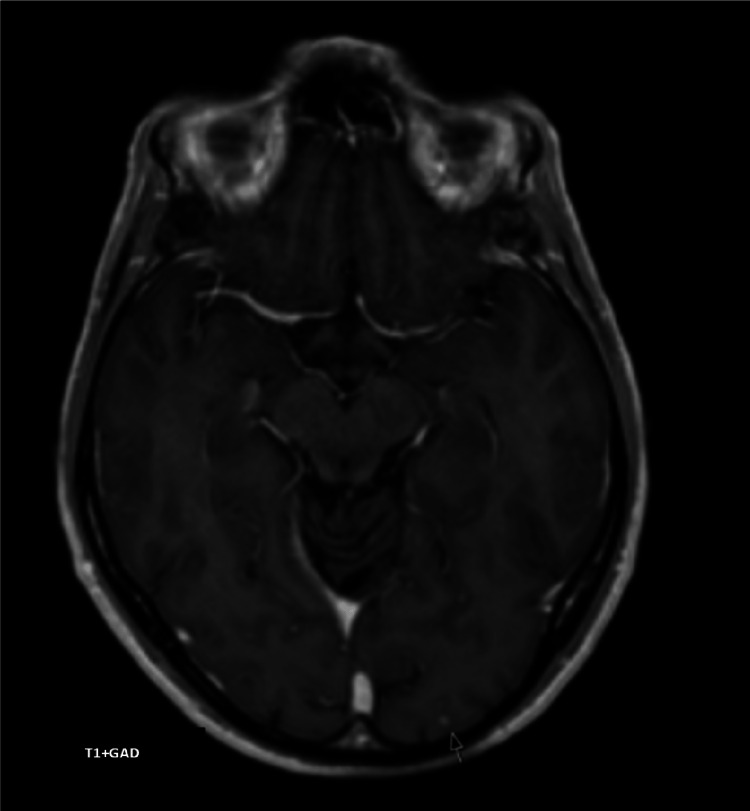
Brain magnetic resonance imaging Lesion with nodular contrast enhancement in the left occipital location (arrow), with a marked reduction in its dimensions compared to the previous image. No halo of vasogenic edema.

He was discharged, with continuing follow-up at the infectious diseases consultation, with the resolution of diplopia within three months after discharge and clear improvement of the imaging alterations described in the control brain MRI nine months after hospitalization, with the disappearance of the lesions located on the sublenticular region on the right and parietal region on the left, as well as the area of associated vasogenic edema; the lesion with nodular contrast uptake in the left occipital location showed a marked reduction in its dimensions, with no halo of vasogenic edema (Figure 2), which ended up supporting the diagnosis of VZV encephalitis. It should also be noted that there was a clear improvement in adherence to ART therapy, with a CD4 cell count of 352/uL at nine months of reassessment.

## Discussion

VZV can affect the central nervous system in different and varied ways and can manifest itself in the form of cerebellar ataxia, arteritis, myelitis, meningitis, and encephalitis. CNS infection can occur during primary infection or during virus reactivation, and the main factors that increase the risk of VZV reactivation are advanced age and immunosuppression [5].

This is an interesting case of acute encephalitis in a patient with an important risk factor for VZV infection, HIV immunosuppression, which evolved favorably after the institution of adequate therapy.

Currently, with the wide availability of ART, the incidence of herpes zoster has been decreasing in patients with HIV, remaining, however, higher than in the general population. The incidence increases the lower the CD4 cell count is, which demonstrates the importance of T-cell-mediated immunity in controlling reinfection, which in the case of the presented patient translates into a high risk of VZV infection due to poor adherence to therapy and consequently poor control of HIV infection [5].

VZV encephalitis is usually manifested by headache, fever, vomiting, altered consciousness, or convulsions. Focal neurological alterations may also occur, such as hemiparesis, cranial nerve paralysis, and pathological osteotendinous reflexes [5,6]. These changes are usually preceded by a vesicular skin rash; however, clinical manifestations may be atypical in immunosuppressed individuals, and skin lesions may be absent, masking the diagnosis of VZV infection in these patients [6]. As is the case with the patient presented, despite being rarer, they have become more frequent due to the improvement and advancement of diagnostic methods, such as the polymerase chain reaction (PCR), which has significantly contributed to better identification in the CSF of many neurotropic viruses capable of causing infectious encephalitis [7].

Although neuroimaging is an important component in the investigation of acute encephalitis, CT often does not demonstrate any alteration, with MRI being the best method for evaluating the brainstem, deep brain structures, and cerebellum and identifying signs of vasculitis. The most common findings are edematous changes with hyperintense areas on the T2 sequence, mainly in the temporal and inferior frontal lobes, with cerebral vasculitis being a frequent complication of VZV encephalitis and stenoses of the cerebral arteries often associated with ischemic lesions of the gray-white matter interface, which is associated with a higher risk of long-term comorbidities [5,7]. In this case, the brain MRI findings are atypical due to the absence of acute ischemic lesions with diffusion restriction, associated with damage to the vascular endothelium. The small lesions described, exhibiting hyperintense signal on T2/FLAIR and homogeneous enhancement after gadolinium, could be secondary to the demyelination process, which has rarely been described in the literature, namely in immunocompromised patients who are affected by VZV infection [8]. At this stage, the differential diagnosis of ischemic lesions in the subacute phase couldn't be absolutely excluded. However, the complete resolution of the lesions in brain MRI follow-up nine months later, after the treatment, revealing the absence of sequelae areas, favors the hypothesis of a non-vascular process, such as demyelination.

CSF analysis is fundamental in the diagnosis of encephalitis, generally demonstrating an increase in the number of leukocytes with a predominance of lymphocytes, proteinorrachia, with a total protein value generally below 150 mg/dl, and a normal or slightly reduced glucose concentration. The detection of VZV DNA in the CSF through the PCR test confirms the diagnosis [5].

Treatment of VZV encephalitis involves the intravenous administration of acyclovir at a dose of 10 to 15 mg/kg every eight hours for a period of seven to 14 days, with a longer period being recommended in immunosuppressed patients. Intravenous administration is preferred since the oral bioavailability of this drug is low [2,5]. In the case presented, the patient completed the 14 days of treatment with intravenous acyclovir, with clinical improvement and improvement of the CNS lesions observed in the imaging exam.

## Conclusions

This case report highlights the clinical presentation, approach, and treatment of an immunosuppressed patient diagnosed with VZV encephalitis. In this case, the authors draw special attention to the importance of the epidemiological context in determining suspicion and diagnosing progress. In this case, we are probably facing a reactivation of VZV in an immunosuppressed patient who does not know whether he had the infection in childhood but who started symptoms before the appearance of chickenpox in his child, with the patient probably being the source of contagion. This case was challenging due to the unusual manifestation of VZV without the appearance of the typical skin lesions, but the diagnosis was confirmed with the identification of VZV DNA in the CSF and corroborated by the clinical improvement and improvement of the lesions observed in the brain MRI with targeted treatment. The authors consider it extremely important to include VZV encephalitis in the differential diagnosis of all CNS manifestations in immunosuppressed patients to avoid delay in diagnosis and initiate therapy as early as possible.
